# Endoscopic treatment of Bouveret syndrome with a combination of electrohydraulic lithotripsy and balloon expansion: A case report

**DOI:** 10.1002/deo2.232

**Published:** 2023-03-27

**Authors:** Kotaro Watanabe, Hirokazu Kawai, Toshifumi Sato, Masaaki Natsui, Ryosuke Inoue, Mayuki Kimura, Kazumi Yoko, Syun‐ya Sasaki, Masashi Watanabe, Yoshihisa Tsukada, Shuji Terai

**Affiliations:** ^1^ Department of Internal Medicine Niigata Prefectural Shibata Hospital Niigata Japan; ^2^ Department of Internal Medicine Niigata Prefectural Kamo Hospital Niigata Japan; ^3^ Department of Gastroenterology Saiseikai Niigata Hospital Niigata Japan; ^4^ Division of Gastroenterology and Hepatology, Graduate School of Medical and Dental Sciences Niigata University Niigata Japan

**Keywords:** balloon, Bouveret syndrome, electrohydraulic lithotripsy, gallstone, pneumonia

## Abstract

Bouveret syndrome is a rare type of ileus caused by the impaction of gallstones passing through a cholecystoenteric fistula in the duodenum. Endoscopic treatment with minimally invasive procedures is preferable for patients with this syndrome, typically for elderly individuals with a high surgical risk. Conventional endoscopic techniques often fail to remove impacted stones that are generally large and occasionally solid. We report the case of an 88‐year‐old bedridden woman with severe dementia who presented with difficulty in breathing. The patient was diagnosed with aspiration pneumonia. In addition, computed tomography showed a cholecystoduodenal fistula and a gallstone 37 mm in diameter that impacted the duodenal bulb. Bouveret syndrome was diagnosed on the basis of the computed tomography findings. The impacted stone was too large and hard to split with standard endoscopic lithotripsy using grasping forceps, mechanical lithotripter, polypectomy snare, basket catheter, and electrohydraulic lithotripsy (EHL). However, EHL with a dual‐channel therapeutic endoscope was achieved to drill a narrow hole approximately 20 mm deep into the stone, in four sessions. The stone was subsequently split by inflating the balloon, which was inserted into the hole, to 10 mm in diameter at 3 atm. All the split stones were spontaneously excreted during defecation after a few days. If the gallstone is too hard to fragment by endoscopic EHL alone, a combination of EHL and balloon expansion might be a useful alternative.

## INTRODUCTION

Bouveret syndrome is a rare type of ileus caused by the impaction of large gallstones that pass through a cholecystoenteric fistula in the duodenum. Gallstone ileus complicates 0.3%–0.5% of patients with cholelithiasis, and Bouveret syndrome constitutes only 1%–3% of this cohort.^1^ Although endoscopic treatment, rather than surgery, is preferable for patients with this syndrome, who are typically older,^2^ some difficulties in endoscopic procedures must be overcome.^3^ Herein, we describe the usefulness of combined endoscopic procedures of electrohydraulic lithotripsy (EHL) and balloon expansion in a case involving lithotripsy of a hard gallstone that could not be fragmented by EHL alone.

## CASE REPORT

An 88‐year‐old chronically bedridden woman with severe dementia was referred to our hospital because of difficulty in breathing and decreased consciousness. Physical examination revealed a low level of consciousness, a Glasgow Coma Scale score of 7, and coarse crackles in the left lung, with copious white sputum. Laboratory tests revealed neutrophilic leukocytosis and an elevated C‐reactive protein level. Chest computed tomography (CT) showed consolidation in the left lower lung field and cholelithiasis with a thickened duodenal wall. The patient was diagnosed with aspiration pneumonia and was hospitalized. The consciousness level and inflammatory indices improved after 3 days of antibiotic treatment.

**FIGURE 1:**
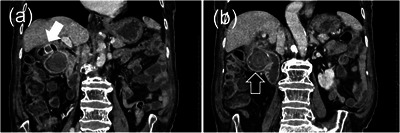
Computed tomography showing calcified stones (white arrow) and gas in the gallbladder with a cholecystoduodenal fistula (a) and a stone (black arrow) in the duodenal bulb (b).

**FIGURE 2:**
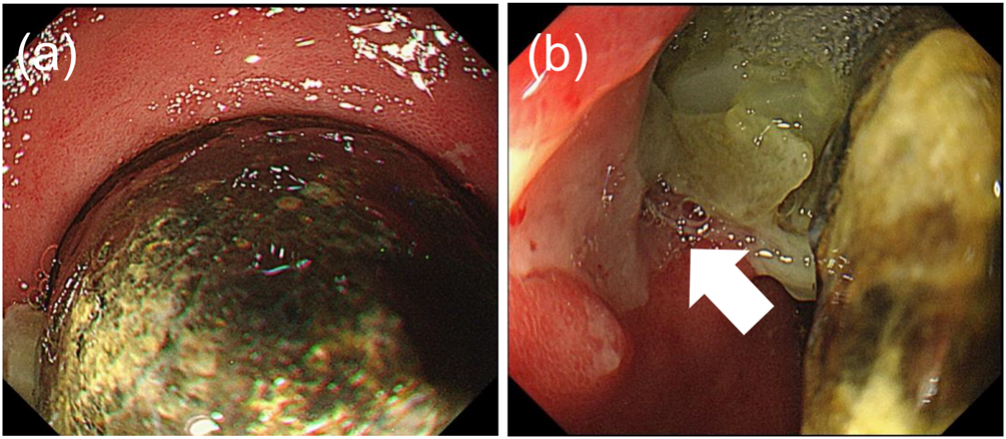
Esophagogastroduodenoscopy showing a stone impacted in the duodenal bulb (a) and a fistula (arrow) in the front wall of the bulb (b).

Contrast‐enhanced abdominal CT on post‐admission day 6 showed calcified stones in the gallbladder and the duodenum, and gas in the gallbladder with a cholecystoduodenal fistula (Figure 1a,b). The stone in the duodenal bulb was 37 × 30 mm in diameter. Esophagogastroduodenoscopy showed a calculus impact in the bulb and a fistula in the front wall of the bulb (Figure 2a,b). On the basis of the CT and esophagogastroduodenoscopy findings, the patient was diagnosed with Bouveret syndrome, causing duodenal obstruction and aspiration pneumonia.

**FIGURE 3:**
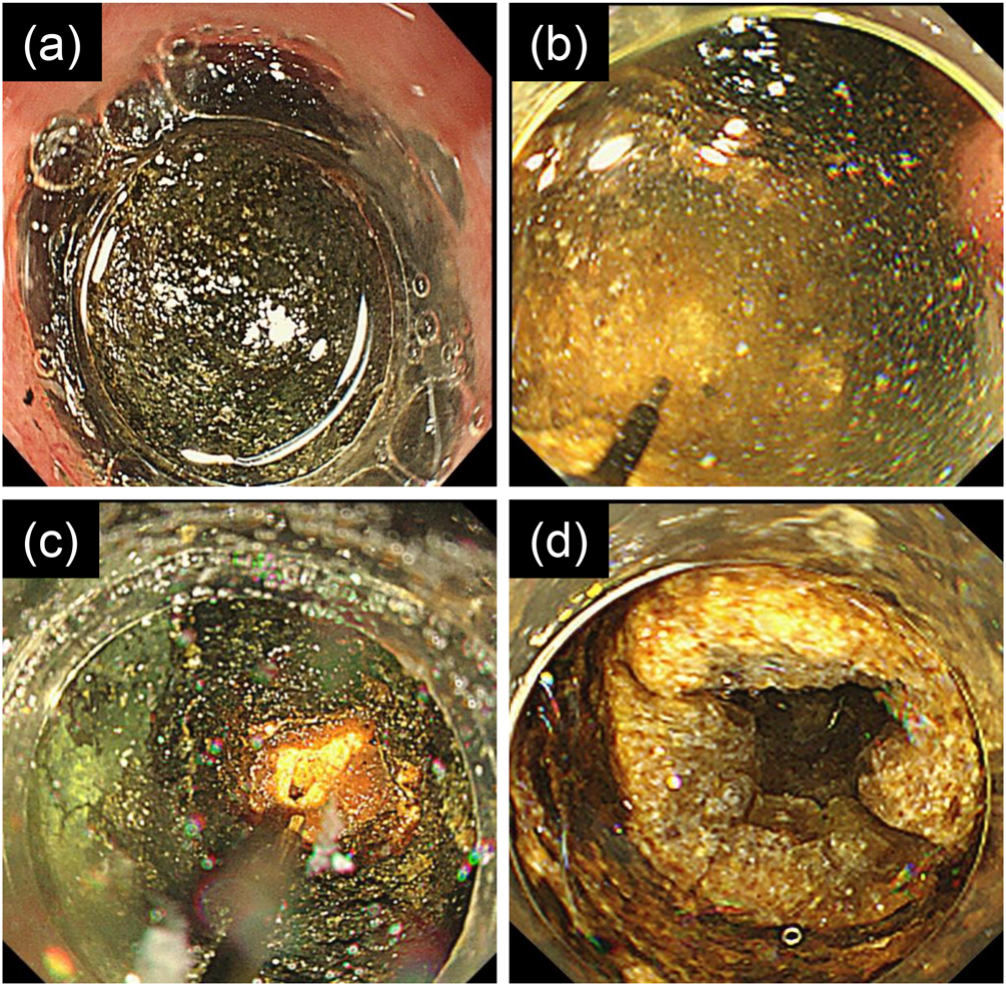
A dual‐channel therapeutic endoscope with a transparent hood showing a hard stone impacted in the duodenal bulb (a). The bulb is filled with normal saline, which is continuously injected via one endoscope channel, and an electrohydraulic lithotripsy (EHL) probe is advanced into the other channel (b). Although the stone surface is shallowly scraped off with EHL, the solid stone cannot be fragmented (c). A narrow hole of approximately 20 mm deep is dug in the stone by repeated EHL (d).

We performed endoscopic procedures rather than surgery to remove the duodenal stone because of the patient's poor performance status and poor respiratory function. However, endoscopic lithotripsy with grasping forceps, a mechanical lithotripter, a polypectomy snare, and a basket catheter could not split the stone because it was too large to be captured by the lithotripter or snare and too hard to be crushed by the grasper. We performed EHL using Autolith (Boston Scientific, Marlborough, MA, USA) with GIF‐2T240 (Olympus, Tokyo, Japan), which is a dual‐channel therapeutic endoscope, attached to a transparent hood (Figure 3a). The bulb was filled with normal saline, which was continuously injected via one endoscope channel, and an EHL probe, passing through a sheath of polypectomy snare, was advanced into the other channel (Figure 3b). The generator settings of EHL were at medium or high power and 10 shots per second. Although the stone surface was shallowly scraped off with EHL, the solid stone could not be fragmented (Figure 3c).

**FIGURE 4:**
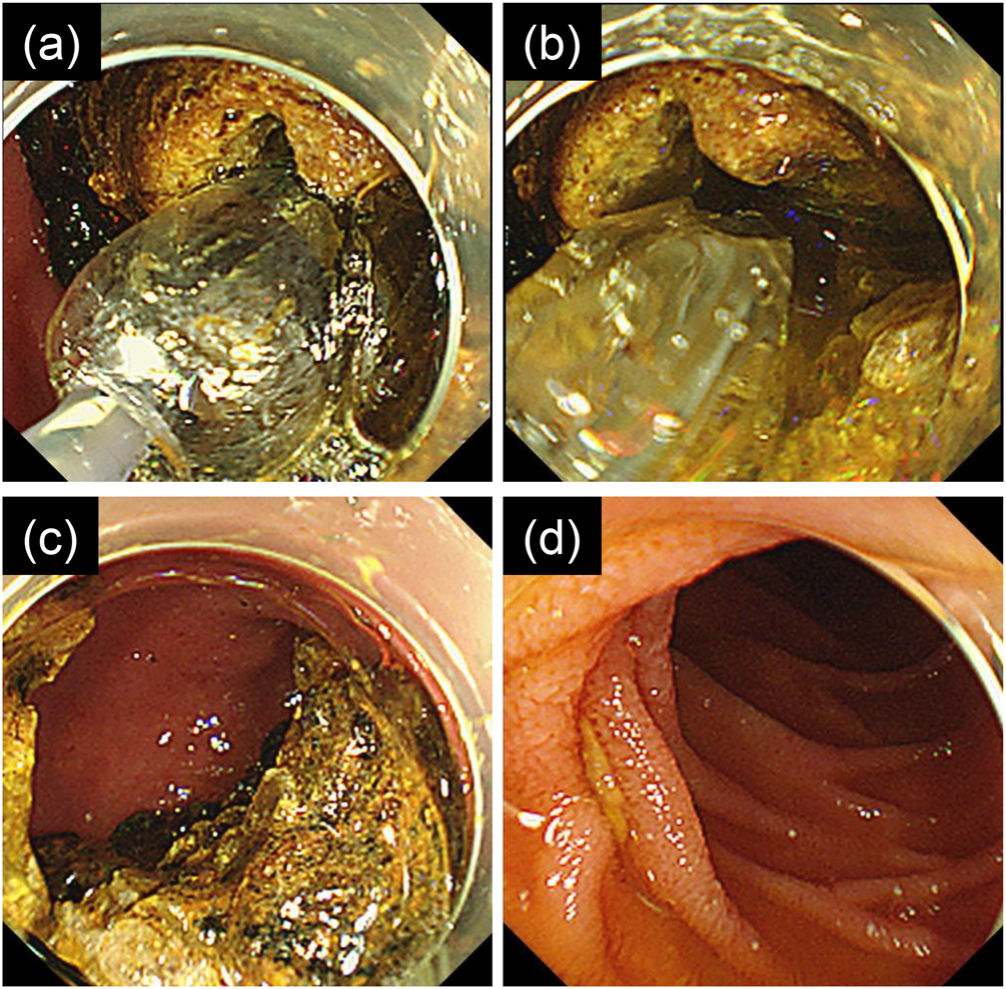
Endoscopic procedures subsequent to digging a hole in the stone. After a biliary balloon dilatation catheter is inserted into the hole of the stone as deeply as possible, the balloon is slowly inflated to 10 mm in diameter at 3 atm (a). The stone is successfully split into two hemispherical pieces (b, c), and both pieces are quickly dislodged into the jejunum (d), providing relief from gastric outlet obstruction.

As EHL alone did not efficiently crush the stone, we attempted lithotripsy with balloon expansion. A narrow hole of approximately 20 mm deep was dug in the stone by the EHL, which needed a total of approximately 14,000 shots in 6 h with four treatment sessions (Figure 3d). The endoscopic procedures were divided into those many sessions from the necessity of tracheal suctioning of copious sputum every hour in the treatment. In the fifth session, a biliary balloon dilatation catheter (CRE^®^ 10–12 mm in balloon diameter, Boston Scientific), whose tip was cut down to advance the balloon into the hole as deeply as possible, was inserted into the hole of the stone. The stone was successfully split into two hemispherical pieces when the balloon was slowly inflated to 10 mm in diameter at 3 atm (Figure 4a–c). Both pieces were quickly dislodged into the jejunum (Figure 4d) and were excreted during defecation after a few days. Chemical analysis of the stones showed cholesterol (95%) and bilirubin calcium (5%) as their components. The patient resumed oral intake and was transferred to a sanatorium.

## DISCUSSION

Bouveret syndrome is characterized by mechanical obstruction of the gastric outlet by duodenal gallstones. It was first reported by Beaussier in 1770 and was redescribed in two case reports by Leon Bouveret^4^ in 1896. A previous review of Bouveret syndrome revealed prominent symptoms including nausea, vomiting, and abdominal pain.^2^ Physical examination usually shows non‐specific signs, including abdominal tenderness, distension, and dehydration. However, in this patient, we initially observed symptoms and signs of aspiration pneumonia, which might be attributed to gastroesophageal reflux due to duodenal obstruction. Therefore, in elderly patients with aspiration pneumonia, which is frequently associated with dysphagia, the gastric outlet should be carefully evaluated to identify the possible underlying diseases.

The prevalence of Bouveret syndrome is higher in older women than that in older men, with an average age of 74 years in women and a female‐to‐male ratio of 1.9.^2^ Patients with older age are often associated with multiple comorbidities, leading to a high mortality rate of 33%.^5^ Thus, an endoscopic approach with minimally invasive procedures should be attempted as first‐line therapy for frail patients with this syndrome.^6^

Various endoscopic modalities, including nets and baskets for stone extraction, snares, forceps, mechanical and laser lithotripsy, and EHL for stone fragmentation, or combinations of these techniques, have been used to relieve gastric outlet obstruction in Bouveret syndrome.^3^ Simple removal with nets and baskets often fails for large stones. Mechanical lithotripsy can be useful for crushing large stones with subsequent fragment extraction. Nevertheless, mechanical lithotripters and snares may be unable to entrap large stones in the narrow duodenal space, as observed in our patient. Among other lithotripsy techniques, EHL is more readily available and less expensive than laser lithotripsy, as Sethi et al. first described in a case report.^7^

Unfortunately, EHL alone could not fragment the gallstone in our patient, because it was extremely hard. We then decided to split the stone using a combination of EHL and balloon expansion. First, a narrow hole was dug into the stone using EHL. Then, the expansion of the balloon within the hole achieved splitting the stone. Although this distinct combination procedure has been also employed by Vanek et al.,^8^ we devised the shortening of the balloon catheter tip by cutting down to prevent a kickback of the balloon. Furthermore, in comparison with Vanek's report, the gallstone might have been more solid in our patient, because EHL could not drill such a large crater in the stones as the authors reported. In addition, our patient was much older than their patient at 59 years and was accompanied by poorer respiratory function with copious sputum. Despite these adverse conditions, we accomplished the lithotripsy in multiple treatment sessions. The combination procedures in multiple sessions may be an alternative method for difficult stones for which EHL alone is ineffective, even in elderly patients with comorbidities.

Endoscopic treatment has several limitations, including a low success rate of approximately 10%^9^ and the need for multiple treatment modalities with high endoscopic expertise.^3^ In addition, distal gallstone ileus due to the migration of fragmented stones can occur.^10^ The removal of all stone fragments after endoscopic lithotripsy is recommended to avoid distal ileus. Close follow‐up was necessary when the fragments were dislodged distally.^5^

In summary, we reported a case of Bouveret syndrome that was successfully managed using a combination of endoscopic EHL and balloon expansion. Although standard management has not been established for Bouveret syndrome, endoscopic treatment is preferable because of its minimal invasiveness, despite being technically challenging. If the gallstone is too hard to fragment by EHL alone, a combination of EHL and balloon expansion may be a useful alternative.

## CONFLICT OF INTEREST STATEMENT

None.

## ETHICS STATEMENT

All procedures were performed in accordance with the ethical standards of the Declaration of Helsinki and its amendments. Written consent was obtained from a patient's proxy.
